# Performance of Enhanced Liver Fibrosis test and comparison with transient elastography in the identification of liver fibrosis in patients with chronic hepatitis B infection

**DOI:** 10.1111/jvh.12161

**Published:** 2013-08-15

**Authors:** P M Trembling, P Lampertico, J Parkes, S Tanwar, M Viganò, F Facchetti, M Colombo, W M Rosenberg

**Affiliations:** 1 Institute for Liver and Digestive Health, Division of Medicine, University College LondonLondon, UK; 2 AM and A Migliavacca Center for Liver Disease, 1st Division of Gastroenterology, Department of Medicine, Fondazione IRCCS Cà Granda Ospedale Maggiore Policlinico, Università degli Studi di MilanoMilan, Italy; 3 Public Health Sciences and Medical Statistics, Faculty of Medicine, University of SouthamptonSouthampton, UK; 4 UO Epatologia, Ospedale San Giuseppe, Università degli Studi di MilanoMilan, Italy

**Keywords:** chronic hepatitis B, Enhanced Liver Fibrosis test, liver fibrosis, noninvasive markers, transient elastography

## Abstract

Assessment of liver fibrosis is important in determining prognosis, disease progression and need for treatment in patients with chronic hepatitis B (CHB). Limitations to the use of liver biopsy in assessing fibrosis are well recognized, and noninvasive tests are being increasingly evaluated including transient elastography (TE) and serum markers such as the Enhanced Liver Fibrosis (ELF) test. We assessed performance of ELF and TE in detecting liver fibrosis with reference to liver histology in a cohort of patients with CHB (*n* = 182), and compared the performance of these modalities. Median age was 46 and mean AST 70 IU/L. Cirrhosis was reported in 20% of liver biopsies. Both modalities performed well in assessing fibrosis at all stages. Area under receiver operator characteristic (AUROC) curves for detecting METAVIR fibrosis stages F ≥ 1, F ≥ 2, F ≥ 3 and F4 were 0.77, 0.82, 0.80 and 0.83 for ELF and 0.86, 0.86, 0.90 and 0.95 for TE. TE performed significantly better in the assessment of severe fibrosis (AUROC 0.80 for ELF and 0.90 for TE, *P* < 0.01) and cirrhosis (0.83 for ELF and 0.95 for TE, *P* < 0.01). This study demonstrates that ELF has good performance in detection of liver fibrosis in patients with CHB, and when compared, TE performs better in detection of severe fibrosis/cirrhosis.

## Introduction

Chronic hepatitis B (CHB) caused by infection with the hepatitis B virus (HBV) is characterized by periods of continuous or fluctuating inflammation of the liver, leading to fibrosis, which may remain occult, with no clinical signs or symptoms at the time of diagnosis of CHB. Morbidity and mortality in patients with CHB are related to persistence of viral replication and the development of liver fibrosis that may progress to cirrhosis and its complications, particularly portal hypertension and liver cancers including hepatocellular cancer, and an increased risk of intra- and extrahepatic biliary cancer 1,2. The assessment of liver fibrosis is therefore an essential component in the initial evaluation of patients with CHB and informs the decision to commence antiviral therapy. Liver fibrosis assessment using invasive or noninvasive tests is a key feature of international guidelines 3,4. Continued monitoring of fibrosis is critical to determine changes in fibrosis over time and to assess the efficacy of therapy and the necessity for interventions to manage portal hypertension and screen for liver cancer and progression to cirrhosis.

The traditional method for assessing liver fibrosis has been needle biopsy of the liver, however this is expensive, frequently painful and potentially hazardous for the patient, and subject to sampling error and variation in interpretation 5,6. While many patients with CHB can be persuaded to undergo a first biopsy, most will be reluctant to accept subsequent follow-up biopsies to evaluate disease progression or response to treatment. Noninvasive methods of assessing liver fibrosis in a range of chronic liver diseases are being explored. Principal among these are transient elastography (TE) and serum markers, and these are now being evaluated in patients with CHB 7–9. The Enhanced Liver Fibrosis (ELF) test (Siemens Healthcare Diagnostics Inc., Tarrytown, New York, USA) is a panel of biomarkers comprising hyaluronic acid (HA), tissue inhibitor of matrix metalloproteinase-1 (TIMP-1) and aminoterminal propeptide of procollagen type III (PIIINP), derived from studies in patients with a range of chronic liver diseases including CHB 10.

Previous studies comparing the performance of noninvasive markers of liver fibrosis in CHB have reported contradictory results. Performance defined by the area under the receiver operator curve (AUROC) of TE to identify F ≥ 2 has been reported in several studies to range from 0.61 to 0.87 11–16.

The aim of this primary study was to evaluate and validate the performance of ELF in a cohort of patients with CHB and to compare ELF to a different noninvasive modality, TE, in the assessment of liver fibrosis defined by histological staging of liver biopsies.

## Materials and Methods

### Patients

Subjects were recruited at a single Italian centre. Among 224 treatment-naïve patients with CHB who were consecutively referred for a liver biopsy and TE evaluation to the Liver Center, Fondazione IRCCS Ca' Granda Ospedale Maggiore Policlinico, Milan 8, those with a stored serum sample available for ELF testing were included. Patients with hepatitis C virus, hepatitis delta virus and human immunodeficiency virus coinfections, other concomitant liver diseases, current or previous hepatic decompensation, current or previous antiviral treatment and/or an absolute contraindication to liver biopsy (platelet count <60 × 10^9^/L, INR > 1.35) were excluded. In all patients, serum sampling, liver biopsy and TE were performed on the same day. All patients gave their written consent to the study, which was approved by the local ethics committee.

### Blood markers

Serum samples were analysed for levels of HA, TIMP-1 and PIIINP using the proprietary assays developed for the ELF test by Siemens Healthcare Diagnostics Inc. These assays are magnetic particle separation immunoassays, and samples were analysed on an ADVIA Centaur® immunoassay system (Siemens Medical Solutions Diagnostics Inc., Tarrytown, NY, USA). Results were entered into the manufacturer's published algorithm to derive an ELF score.

Quantitative polymerase chain reaction amplification for HBV DNA was performed using Amplicor HBV Monitor® (Roche Diagnostics, Branchburg, NJ, USA), and serology for HBeAg status was assessed with standard assays, and serum alanine aminotransferase (ALT) and aspartate transaminase (AST) were measured using standard enzymatic immunoassays.

### Liver biopsy

All patients underwent an ultrasound-guided liver biopsy with a semiautomatic modified Menghini system (16G, BioMol, Hospital Service, Pomezia, Italy, Philips iU22, Bothell, WA, USA) to stage severity of hepatitis. All the procedures were carried out by two highly experienced hepatologists. Liver specimens were considered of adequate size if longer than 2 cm. Patients with a smaller specimen underwent a repeat procedure during the same session. Five-micron thick sections of formalin-fixed, paraffin-embedded liver tissue were stained with haematoxylin–eosin and Masson trichrome, and read by a single liver pathologist blind to TE and clinical data. Grading and staging were evaluated according to METAVIR (staging F0 = fibrosis absent, F1 = portal fibrosis without septa, F2 = portal fibrosis with few septa, F3 = severe fibrosis, F4 = cirrhosis) 17.

### Transient elastography

After an overnight fast, patients underwent a FibroScan® (Echosens, Paris, France) utilizing a 5-MHz ultrasound transducer probe mounted on the axis of a vibrator that was operated by three experienced hepatologists who were blind to clinical, biochemical and histological data 18,19. Briefly, mild amplitude and low-frequency vibrations (50 Hz) are transmitted to the liver, thus inducing an elastic shear wave propagating through the underlying liver tissue. Velocity of the wave is directly related to tissue stiffness. The tip of the transducer was covered with a drop of gel and placed perpendicularly in the intercostal space with the patient lying in dorsal decubitus with the right arm in maximal abduction. Under control time motion and A-mode, the operator chose a liver portion within the right liver lobe at least 6 cm thick, free of large vascular structures and gallbladder. Ten successful acquisitions were performed on each patient. The success rate (SR) was calculated as the ratio of the number of successful acquisitions over the total number of acquisitions. The median value, expressed in kPa, was kept as representative of the liver stiffness. The manufacturer recommends that liver stiffness measurements are considered reliable using the following criteria: (i) number of valid acquisitions at least 10, (ii) SR at least 60% and an interquartile range of the median of 30% or less.

### Statistical analysis

Statistical analyses were performed using SPSS for Windows (version 19, SPSS Inc, Chicago, IL, USA), Stata Statistical Software (StataCorp 2007. Release 10. College Station, TX, USA: StataCorp LP) and R (version 2.11.1, R Foundation for Statistical Computing, Vienna, Austria). Median values and interquartile ranges for each diagnostic test were determined for each fibrosis stage. The diagnostic performances of ELF and TE were assessed by deriving the area under receiver operator characteristic (AUROC) curves. AUROC and 95% confidence intervals of AUROC were calculated. Comparisons of AUROC values for ELF and TE were determined for each stage of fibrosis using the DeLong method to calculate the chi-squared value for the comparison and expressed as the significance of difference (*P* value) 20.

Optimal cut-off values for discriminating positive and negative cases at each fibrosis stage for ELF and TE were determined by identifying the point of maximum sensitivity and specificity on the ROC curve, and sensitivity, specificity, positive and negative predictive values (PPV and NPV), and positive and negative likelihood ratios calculated. The clinical utility of each test was evaluated by analysing performance by selecting an upper threshold with high specificity, therefore high PPV to ‘rule in’ fibrosis and a low threshold with high sensitivity and therefore high NPV to ‘rule out’ fibrosis.

Logistic regression analysis was conducted to further investigate the relationship both between individual modalities and fibrosis, and within a model combining both ELF and TE.

Recently, several methodological issues have been raised in relation to the application of ROC curve analysis to compare noninvasive tests with liver biopsy. The spectrum effect (the differences in the distributions of fibrosis stages in the sample and reference populations) may result in the performance of a noninvasive test varying between the populations giving rise to apparent differences in performance of tests between different sample populations. In addition, ROC analysis assumes the reference standard to be binary, whereas the METAVIR scoring system employs a five-stage ordinal scale. To overcome these potential flaws, the difference between advanced and nonadvanced (DANA) fibrosis stages 21 and Obuchowski 22 methods of correcting for spectrum effect were applied. The results are presented of applying the Obuchowski measure using previously described penalty functions 23 to correct for the degree of difference between the histological stages ascribed by pathological staging and conversion of ELF test scores.

## Results

Of the 224 subjects consecutively recruited, 188 had a stored serum sample. TE acquisition was unsuccessful in six of these subjects (3%); therefore, paired ELF and TE data were available for 182 subjects. Replacing values for missing TE results by both imputation of simple mean and expectation maximization methods did not change the significance of difference between ELF and TE in ROC analysis, therefore, only subjects with paired results were used in the analysis.

Baseline characteristics are shown in Table 1. All patients had a diagnosis of CHB and were treatment-naïve. Median age was 46 years, 71% were male, and 71% were HBeAg negative. 79 (43%) were overweight (BMI ≥ 25 kg/m^2^). Biopsies reported any fibrosis (METAVIR F ≥ 1) in 90.1%, moderate fibrosis (METAVIR F2) in 25.8% and severe fibrosis/cirrhosis (METAVIR F ≥ 3, equivalent to Ishak stage 4–6) in 36.8%.

**Table 1:** Baseline subject characteristics

		By METAVIR stage				
Characteristic	All subjects	F0	F1	F2	F3	F4
Number of subjects	182	18 (9.9)	50 (27.5)	47 (25.8)	31 (17.0)	36 (19.8)
Age, median (range)	46 (18–67)	32.5 (21–54)	44.0 (18–65)	46 (20–67)	55 (27–65)	50 (29–65)
AST (IU/L), mean (SD)	69.7 (64.1)	47.3 (31.9)	49.2 (31.6)	66.4 (38.5)	86.6 (71.1)	97.1 (105.5)
ALT (IU/L), mean (SD)	110.3 (103.4)	86.4 (78.1)	86.7 (72.0)	110.2 (68.0)	148.1 (167.0)	122.6 (112.4)
HBeAg + (n)	53	7	12	10	12	12
− (n)	129	11	38	37	19	24
HBV DNA, log_10_ mean	7.96	7.97	7.82	8.07	7.93	7.98

AST, aspartate transaminase; ALT, alanine aminotransferase; SD, standard deviation; HBV, hepatitis B virus.

**Fig. 1:**
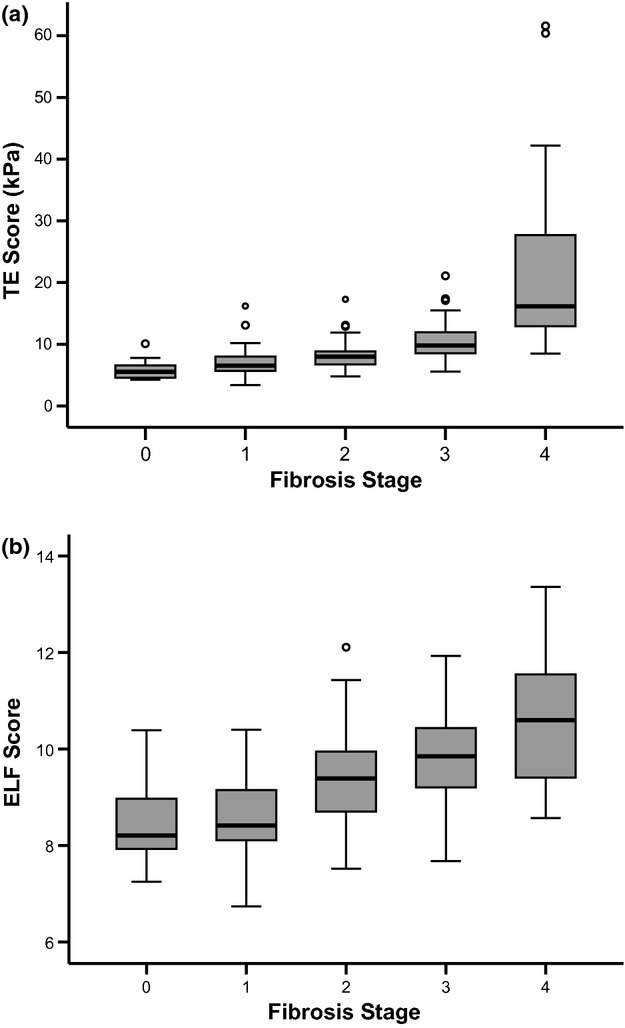
Box plots showing median and quartiles for (a) TE and (b) ELF scores for diagnosing METAVIR fibrosis stages.

Both ELF and TE discriminated different fibrosis stages well with linear progression (Fig. 1), and both modalities performed well in predicting fibrosis stage. The AUROC for the diagnosis of each stage of fibrosis for ELF and TE is shown in Table 2. The AUROC for the diagnosis of any fibrosis for ELF and TE was 0.77 and 0.86, respectively (*P* = 0.09). The AUROC for the diagnosis of severe fibrosis/cirrhosis for ELF and TE was 0.80 and 0.90, respectively (*P* < 0.01). The AUROC for the diagnosis of cirrhosis (METAVIR F4) was 0.83 and 0.95, respectively (*P* < 0.01).

**Table 2:** Median scores and diagnostic performance of ELF and TE according to METAVIR fibrosis stage

	ELF score (*n* = 182)			TE (kPa) (*n* = 182)			
Fibrosis stage	Median (IQR)	AUROC (95% CI)	Adjusted AUROC	Median (IQR)	AUROC (95% CI)	Adjusted AUROC	*P* value*
0 *vs* 1–4	8.21 (1.08) *vs* 9.39 (1.81)	0.77 (0.67–0.87)	0.81	5.55 (2.08) *vs* 8.50 (5.93)	0.86 (0.78–0.94)	0.90	0.09
0,1 *vs* 2–4	8.35 (1.13) *vs* 9.82 (1.53)	0.82 (0.76–0.88)	0.86	6.30 (2.47) *vs* 9.80 (6.43)	0.86 (0.80–0.91)	0.89	0.34
0–2 *vs* 3,4	8.75 (1.35) *vs* 10.06 (1.83)	0.80 (0.73–0.87)	0.83	6.90 (2.60) *vs* 13.00 (11.10)	0.90 (0.85–0.95)	0.94	<0.01
0–3 *vs* 4	9.01 (1.61) *vs* 10.60 (2.16)	0.83 (0.76–0.90)	0.86	7.60 (2.93) *vs* 16.15 (14.77)	0.95 (0.91–0.98)	0.96	<0.01

IQR, interquartile range; AUROC, area under receiver operator characteristic curve; CI, confidence interval.

* Significance of comparison of observed ELF and TE AUROC values.

**Fig. 2:**
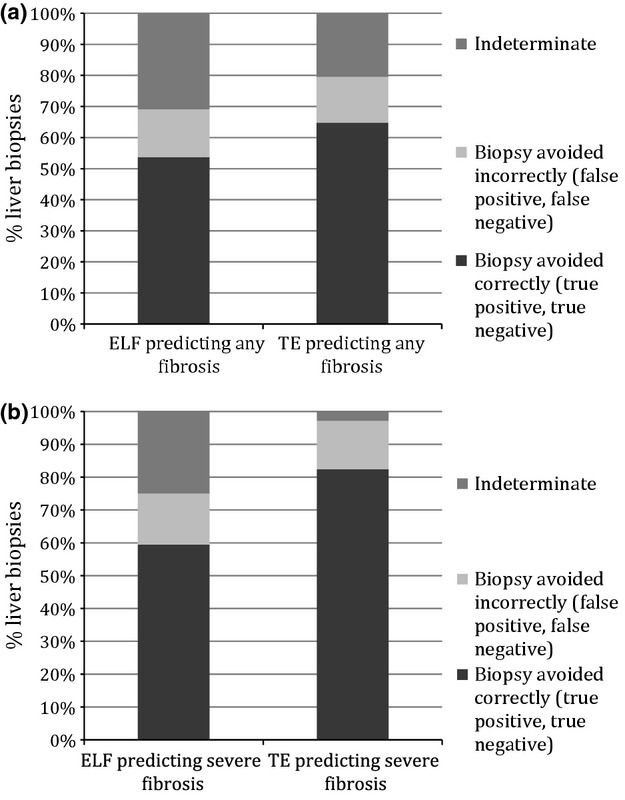
Clinical utility model for ELF and TE predicting (a) any fibrosis and (b) severe fibrosis with sensitivity and specificity of 85%.

Table 3 shows the sensitivities, specificities, predictive values and diagnostic odds ratios of ELF and TE predicting severe fibrosis/cirrhosis and cirrhosis. If two thresholds with high sensitivity and specificity are used to ‘rule in’ fibrosis (upper threshold with high specificity, therefore high positive predictive value) or ‘rule out’ fibrosis (lower threshold with high sensitivity, therefore, high negative predictive value), the clinical utility of each modality can be evaluated. For example, using ELF to identify severe fibrosis at data-derived thresholds of 9.08 and 9.94 (sensitivity and specificity of 85%, respectively), 60% of patients would have correctly avoided liver biopsy and 16% would have incorrectly avoided biopsy. 24% would have had an indeterminate result – a value between the thresholds. Using TE to identify severe fibrosis with thresholds of 8.75 and 8.95 (sensitivity and specificity of 85%, respectively) would have resulted in biopsy correctly being avoided in 82% and incorrectly avoided in 15%, with an indeterminate result in 3%, shown in Fig. 2 and in Table S1. A model for predicting any fibrosis is also shown. At higher sensitivity and specificity, the proportion avoiding biopsy decreases. For example, if sensitivity and specificity thresholds are increased to 90%, the proportion of incorrectly classified cases (i.e. the false positive and false negative rates) substantially decreases to around 10% for both modalities for diagnosis of both severe and any fibrosis. However, this is at the cost of increased proportions of indeterminate cases.

**Table 3:** Diagnostic performance indices for ELF and TE in the identification of severe fibrosis (F3,4) and cirrhosis (F4) at a range of thresholds

Modality	Threshold	Sensitivity%	Specificity%	PPV%	NPV%	LR +	LR −	DOR
Severe fibrosis (prevalence = 37%)								
ELF	8.02	96	17	40	86	1.10	0.24	4.58
	8.45	93	41	48	90	1.58	0.17	9.29
	8.96	85	56	53	86	1.93	0.27	7.15
	9.39	73	70	58	82	2.43	0.39	6.23
	9.88	60	83	67	78	3.53	0.48	7.35
	10.41	45	95	83	75	9.00	0.58	15.52
TE	6.85	96	50	52	95	1.92	0.08	24.00
	7.70	91	60	57	92	2.28	0.15	15.20
	8.45	88	77	69	92	3.83	0.16	23.94
	9.35	79	87	78	88	6.08	0.24	25.33
	10.15	64	90	80	81	6.40	0.40	16.00
	11.95	57	96	88	79	14.25	0.45	31.67
Cirrhosis (prevalence = 20%)								
ELF	8.61	94	39	28	97	1.54	0.15	10.27
	9.43	72	64	34	90	2.00	0.44	4.55
	9.66	69	72	38	90	2.46	0.43	5.72
	9.99	67	81	47	91	3.53	0.41	8.61
	10.34	61	87	54	90	4.69	0.45	10.42
	10.68	44	95	70	87	8.80	0.59	14.92
TE	9.70	94	80	54	98	4.70	0.08	58.75
	10.30	89	86	62	97	6.36	0.13	48.92
	11.85	83	90	67	96	8.30	0.19	43.68
	12.95	75	92	71	94	9.38	0.27	34.74
	14.15	61	95	74	91	12.20	0.41	29.76
	15.45	50	95	72	88	10.00	0.53	18.87

PPV, positive predictive value; NPV, negative predictive value; LR +, positive likelihood ratio; LR −, negative likelihood ratio; DOR, diagnostic odds ratio.

Logistic regression analysis found that in a model combining both modalities, in the prediction of METAVIR F ≥ 1 and F4, ELF was a nonsignificant predictor. In the prediction of F ≥ 2 and F ≥ 3, ELF significantly improved the prediction of fibrosis when combined with TE. Respective ELF and TE odds ratios in the combined models were as follows: 1.45 (95% CI 0.75–2.83) and 1.99 (1.31–3.02), 2.47 (1.55–3.94) and 1.54 (1.25–1.90), 1.61 (1.03–2.51) and 1.55 (1.31–1.83), 1.32 (0.75–2.32) and 1.44 (1.23–1.68) for F ≥ 1, F ≥ 2, F ≥ 3 and F4, respectively (Table S2). Combining the two tests results in AUROC values of 0.87, 0.88, 0.90 and 0.95 for diagnosis of F ≥ 1, F ≥ 2, F ≥ 3 and F4 stages, respectively.

A subanalysis of the performance in HBeAg-negative patients showed similar performance of ELF and TE to that for the whole cohort. AUROC values for ELF and TE for F ≥ 1, F ≥ 2, F ≥ 3 and F4 stages were 0.71, 0.80, 0.79, 0.81 and 0.81, 0.83, 0.90, 0.95, respectively, with a significant difference in performance at F ≥ 3 and F4.

The effect of ALT on test performance was assessed. Diagnostic accuracy appears to be maintained with both modalities when ALT is 3 or 5 times above the upper limit of normal (ULN). In the diagnosis of severe fibrosis, both modalities maintained their performance in all categories of ALT. The AUROC values indicate that in the diagnosis of any fibrosis, ELF is less accurate when ALT is below the ULN compared with when ALT is above the ULN, and accuracy of TE improves when ALT is below the ULN. The 95% confidence interval for ELF in diagnosing any fibrosis is very large in this small cohort. When ALT is above 3 or 5 times the ULN, diagnostic accuracy appears to be maintained with both modalities (Table S3).

## Discussion

This study has demonstrated that the ELF test accurately assesses liver fibrosis severity in patients with CHB. Comparison of TE and ELF demonstrated good performance of both modalities, with TE performing significantly better in the identification of severe fibrosis/cirrhosis.

The ELF test has been validated in external disease-specific cohorts of patients with nonalcoholic fatty liver disease, primary biliary cirrhosis and chronic hepatitis C 24–29. It predicts liver-related outcomes at 7 years at least as well as biopsy, with a unit change in ELF associated with a doubling of risk 30. Of the 25 patients with CHB followed up for over 7 years in that study, 2 died of a liver-related cause and one experienced a nonfatal liver-related outcome by 7 years (median for the whole cohort) after biopsy and ELF test. In all 3 cases, the incident ELF score exceeded 7.8. The median ELF score was 8.63 for the whole cohort of CHB patients that were followed up.

This study reports the external validation of the ELF test in subjects with CHB. Performance in patients with CHB in the original cohort (*n* = 44) was good at all fibrosis stages and maintained in this validation cohort. Logistic regression, which included age and simple biochemical parameters (AST, ALT), did not improve performance. These data suggest a role for ELF in the assessment of patients with CHB and in informing the decision-making process when antiviral therapy is being considered.

A recent study 31 reporting the performance of ELF in 58 patients with CHB used the published algorithm 24 but not the immune assays that have been specifically developed for the ELF test. AUROC values for predicting Ishak fibrosis stages 1–6 and 2–6 (equivalent to METAVIR F ≥ 1) were 0.66 and 0.59, respectively, lower than the values we report. AUROC for predicting Ishak stages 3–6 was 0.83, similar to our findings. The inferior performance of the test in this cohort is likely to be attributable to the use of assays that were not specifically developed for the ELF test and failure to use the appropriate autoanalyser.

Recently, the performance of ELF and TE has been studied in a cohort of Asian subjects with CHB 32. AUROC values for predicting F ≥ 2, F ≥ 3 and F4 were 0.90, 0.86 and 0.86 for ELF and 0.94, 0.96 and 0.96 for TE, respectively. TE was significantly better than ELF for predicting F ≥ 3 and F4.

In the present study, TE performed as well or better than in other studies in patients with CHB. For example, in the detection of F4 fibrosis, AUROC values in other studies range from 0.88 11 to 0.94 33. A meta-analysis of noninvasive tests for liver disease severity in nonalcoholic fatty liver disease 34 found that the collective performance of TE in detecting F ≥ 2 and F ≥ 3 fibrosis was 0.84 (95% CI 0.79–0.90) and 0.94 (95% CI 0.86–0.99), respectively. Regression analysis found that success was unaffected by the severity of inflammation or steatosis, but obesity was an independent predictor of failure of TE.

The rate of TE failure (3%) was very low in this study; a major review of clinical performance found a failure rate of 18.9% 35. Studies investigating TE in patients with CHB report success rates for acquiring valid TE results ranging between 79% and 99.6% 11,16,35–39. TE reproducibility has been shown to be excellent for both inter-and intraobserver agreement, but this is reduced at lesser stages of fibrosis and in patients with steatosis, high body mass index and in particular waist circumference 40,41. All 6 patients in our study excluded due to TE failure were overweight (*n* = 2) or obese (*n* = 4).

Both ELF and TE represent alternative and potentially complimentary approaches to assessing liver fibrosis and are associated with minimal discomfort and hazard to the patient when compared with biopsy. Logistic regression analysis suggests that the performance of ELF is improved with the addition of TE, although TE does not improve with the addition of ELF.

Both modalities track fibrosis stage linearly, with TE having superior discrimination and closer correlation with histological staging, particularly at higher fibrosis stages. The performance of TE predicting F ≥ 2 fibrosis in this study was superior to most of the previous studies assessing TE in CHB. The diagnostic performance of each modality was evaluated at various sensitivities and specificities; the median diagnostic odds ratio for ELF for detecting severe fibrosis between sensitivity and specificity of 95% was 7.3 and for TE 24.0. Clinical utility modelling supports a role for these modalities in the assessment of patients and in treatment decisions.

Applying previously published thresholds to our data allows for some generalizability of the model. Recent studies investigating ELF and TE both in a heterogeneous population 42 and in CHB 32 did not report dual thresholds, making comparison difficult. However, using thresholds reported in separate studies allows some comparisons to be drawn. A study of TE in CHB 8 reported that cut-off values of 9.4 and 6.2 which had sensitivity and specificity of >90% ruled in and ruled out F ≥ 2 in 56% of cases, with 90% accuracy. Applying these thresholds to our data, 57% of patients would have F ≥ 2 ruled in or ruled out, with 91% accuracy. Data from patients with chronic hepatitis C 27 found that using ELF cut-off values of 9.59 and 10.22, with sensitivity and specificity of 85%, 81% of patients could avoid biopsy by having severe fibrosis (F ≥ 3) ruled in or ruled out, with 81% accuracy. Applying these thresholds to our data, 77% of patients would avoid biopsy, with 86% accuracy.

Using the DANA method to calculate the adjusted uniform AUROC, diagnostic performance increased at all fibrosis stages with both modalities. This method assumes equal prevalence in all fibrosis stages, which may not be reflective of true prevalence and may overestimate prevalence at the extremes of fibrosis stage. Further, the coefficient in the equation was developed using a population of patients with chronic hepatitis C, and with a different noninvasive test, although it has been employed subsequently in a cohort of CHB patients 43. Further validation of this method is required. Adjustment using the Obuchowski method showed that the overall mean accuracy (unweighted Obuchowski measure) was 0.91 for ELF and 0.95 for TE. For diagnosis between F3 and F4, performance was 0.59 for ELF and 0.73 for TE (Table S4).

Strengths of this study include the method of data collection. Liver biopsy, TE and serum sampling were all performed on the same day. ELF tests were performed in one central laboratory, ensuring quality control and consistency. It is important to note that the present study used the proprietary ELF assays in accordance with the manufacturer's instructions rather than a ‘homebrew’ combination of substitute assays performed on other platforms as reported in other studies 31. There are several potential limitations to this study. The low failure rate of TE in this study was at odds with much larger reports of clinical practice. The relatively high prevalence of fibrosis in this cohort means that the findings may not be reliably applied to lower prevalence populations such as the primary care setting, where the positive predictive value of the test will be lower.

This study has demonstrated that the performance of ELF in detection of liver fibrosis in subjects with CHB is good and is reproducible. Both ELF and TE perform well in the prediction of fibrosis at all stages, with TE superior at detecting severe fibrosis and cirrhosis in this cohort that contained a high prevalence of severe fibrosis. Further analyses in cohorts of subjects with CHB are required.

## Conflicts of Interest

Pietro Lampertico has speaker bureau roles for Roche, Gilead, Bristol-Myers Squibb and GlaxoSmithKline. Massimo Colombo has served as an advisory committee member for Merck, Roche, Novartis, Bayer, Bristol-Myers Squibb, Gilead Sciences, Tibotec, Vertex, Janssen Cilag and Achillion. He has served as a speaker and teacher for Tibotec, Roche, Novartis, Bayer, Bristol-Myers Squibb, Gilead Sciences and Vertex and has received grant and research support from Merck, Roche, Bristol-Myers Squibb and Gilead Sciences. William Rosenberg is CEO of iQur Ltd and receives grant funding form Siemens Healthcare Diagnostics Inc. He has speaker bureau roles for Roche and Gilead and is an advisory committee member for Roche, Gilead Sciences, MSD and GlaxoSmithKline. Paul Trembling and Sudeep Tanwar have received educational grant support from Janssen, MSD, Gilead Sciences, Novartis and Roche.

## Abbreviations

ALT: alanine aminotransferase
AST: aspartate transaminase
AUROC: area under receiver operator characteristic curves
CHB: chronic hepatitis B
DANA: difference between advanced and nonadvanced fibrosis stages
ELF: Enhanced Liver Fibrosis test
HA: hyaluronic acid
HBV: hepatitis B virus
NPV: negative predictive value
PPV: positive predictive value
SR: success rate
TE: transient elastography
ULN: upper limit of normal

## SUPPORTING INFORMATION

**Table S1.** Sensitivities and specificities of ELF and TE identifying severe fibrosis (METAVIR ≥ F3) and any fibrosis (F ≥1) using thresholds with sensitivity and specificity of 85%. **Table S2.** Odds ratios calculated by logistic regression for prediction of fibrosis using models comprising ELF, TE and ELF and TE. **Table S3.** Diagnostic performance of ELF and TE according to categories of ALT. **Table S4.** Obuchowski measures for ELF and TE for each fibrosis stage pair.
