# Supplementary material for: Performance of Enhanced Liver Fibrosis test and comparison with transient elastography in the identification of liver fibrosis in patients with chronic hepatitis B infection
Source: J Viral Hepat. 2013 Aug 15;21(6):430–8. doi: 10.1111/jvh.12161 (PMC4298014; doi:10.1111/jvh.12161)
Supplement: Supplementary file 1 [file jvh0021-0430-sd1.docx]

**SUPPLEMENTARY MATERIAL**

**Table 1.** Sensitivities and specificities of ELF and TE identifying severe fibrosis (METAVIR F≥3) and any fibrosis (F≥1) using thresholds with sensitivity and specificity of 85%.

| Fibrosis stage  (Prevalence) | Modality | Thresholds | Sensitivity  (%) | Specificity  (%) | Correctly avoided  (%) | Incorrectly avoided  (%) | Indeterminate  (%) |
| --- | --- | --- | --- | --- | --- | --- | --- |
| Severe fibrosis  (37%) | ELF | 9.08 | 84 | 61 | 60 | 16 | 24 |
|  |  | 9.94 | 57 | 85 |  |  |  |
|  | TE | 8.75 | 85 | 86 | 82 | 15 | 3 |
|  |  | 8.95 | 82 | 85 |  |  |  |
| Any fibrosis  (90%) | ELF | 8.22 | 85 | 50 | 54 | 15 | 31 |
|  |  | 9.24 | 54 | 83 |  |  |  |
|  | TE | 6.15 | 85 | 65 | 65 | 15 | 20 |
|  |  | 7.70 | 64 | 89 |  |  |  |

**Table 2.** Odds ratios calculated by logistic regression for prediction of fibrosis using models comprising ELF, TE and ELF and TE

| **Fibrosis stage** | | **Model** | | | |
| --- | --- | --- | --- | --- | --- |
|  |  | **ELF** | **TE** | **Combined** | |
|  |  |  |  | **ELF** | **TE** |
| 0 *vs* 1-4 | **OR** | 2.58 | 2.14 | 1.45 | 1.99 |
|  | **95% CI** | 1.45-4.60 | 1.44-3.19 | 0.75-2.83 | 1.31-3.02 |
|  | ***P* value** | 0.001 | <0.001 | 0.27 | 0.001 |
|  | **R^2^** | 0.16 | 0.32 | 0.33 | |
| 0,1 *vs* 2-4 | **OR** | 3.75 | 1.75 | 2.47 | 1.54 |
|  | **95% CI** | 2.45-5.75 | 1.43-2.15 | 1.55-3.94 | 1.25-1.90 |
|  | ***P* value** | <0.001 | <0.001 | <0.001 | <0.001 |
|  | **R^2^** | 0.39 | 0.44 | 0.53 | |
| 0-2 *vs* 3,4 | **OR** | 2.85 | 1.64 | 1.61 | 1.55 |
|  | **95% CI** | 2.02-4.04 | 1.40-1.93 | 1.03-2.51 | 1.31-1.83 |
|  | ***P* value** | <0.001 | <0.001 | 0.04 | <0.001 |
|  | **R^2^** | 0.34 | 0.56 | 0.58 | |
| 0-3 *vs* 4 | **OR** | 3.00 | 1.49 | 1.32 | 1.44 |
|  | **95% CI** | 2.04-4.43 | 1.30-1.72 | 0.75-2.32 | 1.23-1.68 |
|  | ***P* value** | <0.001 | <0.001 | 0.34 | <0.001 |
|  | **R^2^** | 0.36 | 0.62 | 0.62 | |

OR, odds ratio; CI, confidence intervals; R^2^, Nagelkerke pseudo R-square values

**Table 3.** Diagnostic performance of ELF and TE according to categories of ALT

| ALT category | Modality | AUROC (95% CI) | | | |
| --- | --- | --- | --- | --- | --- |
|  |  | Fibrosis stage | | | |
|  |  | 0 *vs* 1-4 | 0,1 *vs* 2-4 | 0-2 *vs* 3,4 | 0-3 *vs* 4 |
| <ULN  n=24 | ELF | 0.51  (0.06-0.96) | 0.86  (0.66-1.00) | 0.81  (0.62-1.00) | 0.89  (0.76-1.00) |
|  | TE | 0.94  (0.85-1.00) | 0.95  (0.87-1.00) | 0.92  (0.82-1.00) | 1.00  (1.00-1.00) |
| >ULN  n=158 | ELF | 0.81  (0.76-0.94) | 0.82  (0.76-0.89) | 0.80  (0.72-0.87) | 0.82  (0.74-0.90) |
|  | TE | 0.85  (0.76-0.94) | 0.84  (0.78-0.90) | 0.90  (0.85-0.95) | 0.94  (0.90-0.98) |
| >3xULN  n=55 | ELF | 0.85  (0.70-1.00) | 0.83  (0.72-0.94) | 0.81  (0.69-0.92) | 0.87  (0.77-0.97) |
|  | TE | 0.99  (0.97-1.00) | 0.79  (0.65-0.93) | 0.90  (0.81-0.98) | 0.95  (0.89-1.00) |
| >5xULN  n=30 | ELF | 0.82  (0.63-1.00) | 0.83  (0.68-0.98) | 0.78  (0.61-0.95) | 0.88  (0.75-1.00) |
|  | TE | 1.00  (1.00-1.00) | 0.83  (0.64-1.00) | 0.87  (0.74-1.00) | 0.97  (0.89-1.00) |

ALT, alanine transaminase; AUROC, area under receiver operator characteristic curve; CI, confidence interval; ULN, upper limit of normal range

**Table 4.** Obuchowski measures for ELF and TE for each fibrosis stage pair

| Fibrosis Stage Pair | ELF | | TE | |
| --- | --- | --- | --- | --- |
|  | Estimate | Standard Error | Estimate | Standard Error |
| 1 *vs* 2 | 0.58 | 0.08 | 0.71 | 0.07 |
| 1 *vs* 3 | 0.80 | 0.06 | 0.87 | 0.06 |
| 1 *vs* 4 | 0.82 | 0.06 | 0.94 | 0.04 |
| 1 *vs* 5 | 0.94 | 0.03 | 0.99 | 0.01 |
| 2 *vs* 3 | 0.74 | 0.05 | 0.70 | 0.05 |
| 2 *vs* 4 | 0.79 | 0.06 | 0.86 | 0.04 |
| 2 *vs* 5 | 0.92 | 0.03 | 0.98 | 0.01 |
| 3 *vs* 4 | 0.59 | 0.07 | 0.73 | 0.06 |
| 3 *vs* 5 | 0.78 | 0.05 | 0.96 | 0.02 |
| 4 *vs* 5 | 0.69 | 0.06 | 0.85 | 0.05 |
| Overall | 0.91 | 0.01 | 0.95 | 0.01 |
